# High-resolution computed tomographic (HRCT) image series from 413 canid and 18 felid skulls

**DOI:** 10.1038/s41597-024-03572-x

**Published:** 2024-07-16

**Authors:** Kalman Czeibert, Gergely Nagy, Tibor Csörgő, Tamás Donkó, Örs Petneházy, Ádám Csóka, László Zsolt Garamszegi, Niclas Kolm, Eniko Kubinyi

**Affiliations:** 1 Department of Ethology, Institute of Biology, ELTE Eötvös Loránd University, Budapest, Hungary; 2 LimesVet Ltd., Budapest, Hungary; 3 Institute of Ecology and Botany, HUN-REN Centre for Ecological Research, Vácrátót, Hungary; 4 Department of Genetics, Institute of Biology, ELTE Eötvös Loránd University, Budapest, Hungary; 5 Medicopus Nonprofit Ltd., Kaposvár, Hungary; 6 Hungarian University of Agriculture- and Life Sciences, Institute of Physiology and Nutrition, Department of Physiology and Animal Health, Kaposvár Campus, Kaposvár, Hungary; 7 Department of Zoology, Stockholm University, Stockholm, Sweden; 8 MTA-ELTE Lendület “Momentum” Companion Animal Research Group, Budapest, Hungary; 9 ELTE NAP Canine Brain Research Group, Budapest, Hungary

**Keywords:** Animal physiology, Brain

## Abstract

Computed tomography (CT) is a non-invasive, three-dimensional imaging tool used in medical imaging, forensic science, industry and engineering, anthropology, and archaeology. The current study used high-resolution medical CT scanning of 431 animal skulls, including 399 dog skulls from 152 breeds, 14 cat skulls from 9 breeds, 14 skulls from 8 wild canid species (gray wolf, golden jackal, coyote, maned wolf, bush dog, red fox, Fennec fox, bat-eared fox), and 4 skulls from 4 wild felid species (wildcat, leopard, serval, caracal). This comprehensive and unique collection of CT image series of skulls can provide a solid foundation not only for comparative anatomical and evolutionary studies but also for the advancement of veterinary education, virtual surgery planning, and the facilitation of training in sophisticated machine learning methodologies.

## Background & Summary

Before the advent of 3D imaging techniques, researchers had to physically visit various collections and handle vulnerable objects to examine their pertinent attributes closely. The adoption of digitized image repositories has dramatically improved access to large samples of specimens from collections, allowing researchers to conduct examinations from their workstations. This capability enables the conducting of analyses on a larger scale than previously undertaken, fostering collaboration and facilitating data sharing among different institutions, thereby expanding the scope of research^1,2^. Consequently, specimens carefully preserved in natural history or private collections can be made accessible to a diverse range of interested scholars^3^. Moreover, digital technologies (e.g., the estimation of cranial cavity volume using glass beads or water is substituted with digital endocasting) may replace traditional methods, offering a more accurate and secure means of inspection^4,5^.

Computed tomography (CT) stands out as a widely employed technique for digitizing specimens. CT, a potent three-dimensional imaging technique, enables non-invasive visualization of internal structures^6^. In veterinary medicine, CT scans are particularly useful for detecting various conditions such as diverse orthopedic conditions (like fractures, arthrosis, congenital disorders), nasal diseases, head traumas, lung diseases, middle and inner ear disorders, dental diseases or certain tumors^7^. Due to its non-destructive nature, CT can be a useful and indispensable tool for digitizing vulnerable specimens in natural science collections^3,8^. It is important to be aware of the difference between the nature of CT and magnetic resonance imaging (MR), as CT is superior in analyzing osseous structures, while MR is particularly useful for examining soft tissue characteristics.

The skull, recognized as the most intricate bone in the body, exhibits substantial variation in form across species and breeds, encompassing a wide range in thickness and density (e.g., from the teeth and petrosal bone to the delicate ethmoturbinates). Evolutionary and comparative studies can leverage characteristic craniometric measurements to illustrate changes in specific areas (e.g., maxillofacial region, nasal cavity, the paranasal sinus system, teeth arrangement, depth, and angle of the orbital, inner and outer neurocranial properties)^9–11^.

Having gained unique access to a private collection comprising hundreds of individual carnivore skulls, we conducted meticulous CT scans on 431 canid and felid species. This dataset holds multifaceted utility, such as our exploration of digital endocasting^12^ and investigation into brain size evolution during domestication among dog breeds^13^. This unique collection of high-quality CT scans of canid and felid skulls is of interest to many scientific or educational fields, and its wide-ranging potential makes it worth sharing with a knowledgeable audience.

The extensive collection of skulls at our disposal presents a valuable opportunity to investigate the anatomical variations among canids and felids, both within and between breeds or species^14–18^, or certain cranial regions^19,20^ (e.g., for comparative morphometrics or to test evolutionary hypotheses). Furthermore, the particular set of species and breeds involved allows for examining the implications of domestication^13,21^. By exploring prospects in education, the usability of our CT images can be extended beyond the research domain. The application of CT images, stored in appropriate medical file formats, provides a robust foundation for 3D visualization, segmentation, and even 3D printing while serving as a basis for developing deep learning methods and algorithms^22,23^. According to Silvera *et al*.^24^, incorporating interactive virtual 3D skull models and skull replicas can prove invaluable in veterinary anatomy education.

## Methods

### Subjects

A total of 431 skulls were scanned, including 399 skulls from 152 dog (*Canis familiaris*) breeds, 14 skulls from 9 cat (*Felis catus*) breeds, 14 skulls from 8 wild canid species (*Otocyon megalotis*, *Speothos venaticus*, *Canis latrans, Vulpes zerda, Canis aureus, Canis lupus, Chrysocyon brachyurus, Vulpes vulpes*), and 4 skulls from 4 wild felid species (*Caracal caracal, Panthera pardus, Leptailurus serval, Felis silvestris*) (Table 1).

**Table 1:** List of CT-scanned skulls.

Species	CT-scanned skulls N
Bat-eared fox (*Otocyon megalotis*)	1
Bush dog (*Speothos venaticus*)	1
Caracal (*Caracal caracal*)	1
Cat (9 breeds, *Felis catus*)	14
Coyote (*Canis latrans*)	1
Dog (152 breeds, *Canis familiaris*)	399
European wildcat *(Felis silvestris)*	1
Fennec fox (*Vulpes zerda*)	1
Golden Jackal (*Canis aureus*)	4
Gray wolf (*Canis lupus*)	3
Leopard (*Panthera pardus*)	1
Maned wolf (*Chrysocyon brachyurus*)	1
Red fox (*Vulpes vulpes*)	2
Serval (*Leptailurus serval*)	1
**Total N**	**431**

The list of the dog breeds (1–9 skulls/breed, see Table S1): Affenpinscher, Afghan Hound, Airedale Terrier, Akita Inu, Alaskan Malamute, American Bulldog, American Pit Bull Terrier, Australian Shepherd, Australian Terrier, Bandog, Basenji, Basset Hound, Beagle, Beauceron (Beauce Sheep Dog), Bedlington Terrier, Belgian Shepherd Dog (Groenendael), Belgian Shepherd Dog (Tervueren), Berger Picard (Picardy shepherd), Bernese Mountain Dog (Berner Sennenhund), Bichon Frisé, Black Russian Terrier, Bolognese, Border Collie, Border Terrier, Borzoi, Boston Terrier, Boxer, Briard, Brittany (French Brittany), Bucovina Shepherd Dog (Southeastern European Shepherd), Bull Terrier, Bulldog, Bullmastiff, Cairn Terrier, Canadian Eskimo Dog, Canary Mastiff (Perro de Presa Canario), Cane Corso, Caucasian Shepherd Dog, Cavalier King Charles Spaniel, Central Asian Shepherd Dog (Ovtcharka), Chihuahua, Chinese Crested Dog, Chow Chow, Collie (Rough), Coton de Tulear, Czechoslovakian Wolfdog, Dachshund (Short-haired), Dachshund (Wire-haired), Dalmatian, Doberman Pinscher, Dogo Argentino, Dogue de Bordeaux, English Cocker Spaniel, English Mastiff, English Pointer, English Setter, English Springer Spaniel, Fila Brasileiro, Fox Terrier (Smooth), Fox Terrier (Wire-haired), French Bulldog, French Pointing Dog (Braque Francais, type Gascogne), German Pointer (Wire-haired), German Shepherd Dog (Alsatian), German Shepherd Dog, Long-haired (Alsatian, Long-haired; Old German Shepherd Dog), German Spaniel, Giant German Spitz, Giant Schnauzer, Golden Retriever, Gordon Setter, Grand Anglo-Français Blanc et Noir, Great Dane, Great Pyrenees (Pyrenean Mountain Dog), Greater Swiss Mountain Dog, Greenland Dog, Greyhound, Griffon Bruxellois, Hanover Hound, Havanese, Hovawart, Hungarian Greyhound, Irish Setter, Irish Wolfhound, Italian Greyhound, Jack Russell Terrier, Japanese Chin, Keeshond (Wolfspitz), Kerry Blue Terrier, King Charles Spaniel, Komondor, Kuvasz, Labrador Retriever, Landseer, Large Münsterländer, Leonberger, Lhasa Apso, Maltese, Medium German Spitz, Medium Poodle, Miniature Bull Terrier, Miniature German Spitz, Miniature Pinscher, Miniature Poodle, Miniature Schnauzer, Moscow Watchdog, Mudi, Neapolitan Mastiff (Mastino Napoletano), Newfoundland, Norwegian Hound (Dunker), Old English Sheepdog (Bobtail), Papillon (Continental Toy Spaniel), Pekingese, Peruvian Hairless Dog, Polish Hound, Pomeranian, Portuguese Sheepdog (Cão da Serra de Aires), Pug, Puli, Pumi, Pyrenean Mastiff, Rhodesian Ridgeback, Rottweiler, Saluki, Samoyed, Schapendoes (Dutch Sheepdog), Scottish Deerhound, Scottish Terrier, Serbian Hound, Shar Pei, Shetland Sheepdog, Shih Tzu, Siberian Husky, Small Münsterländer, Spanish Sighthound (Galgo Español), St. Bernard, Staffordshire Bull Terrier, Standard Poodle, Standard Schnauzer, Tibetan Mastiff, Tibetan Terrier, Tosa, Transylvanian Hound, Vizsla, Weimaraner, Welsh Corgi (Cardigan), Welsh Corgi (Pembroke), Welsh Terrier, West Highland White Terrier, Whippet, White Swiss Shepherd Dog (Berger Blanc Suisse, White German Shepherd, White Shepherd), Yorkshire Terrier, Yugoslavian Shepherd Dog (Šarplaninac).

The list of cat breeds (1-2 skulls/breed, Table S1): Abyssinian, Birman, British shorthair, European shorthair, Himalayan, Maine coon, Scottish fold, Siamese, Sphynx.

### The collection of skulls

The skulls are part of a collection of author TC. He obtained the skulls through private donations from cadavers over the past decades for educational purposes in teaching anatomy. Later, as his collection of skulls evolved, he began to systematically look for rare breeds and species. The animals had died of old age or disease or were euthanized by their owner for medical reasons, irrespective of the present study. Skulls were macerated, degreased, and dried out. Each skull has a unique identifier based on which the species, the breed (and, if it was known, the sex) can be accessed (Table S1). The age of the animals was unknown, but the ossification of their cranial sutures and the eruption of the permanent teeth confirmed that they were adult individuals.

### Image acquisition

**Fig. 1:**
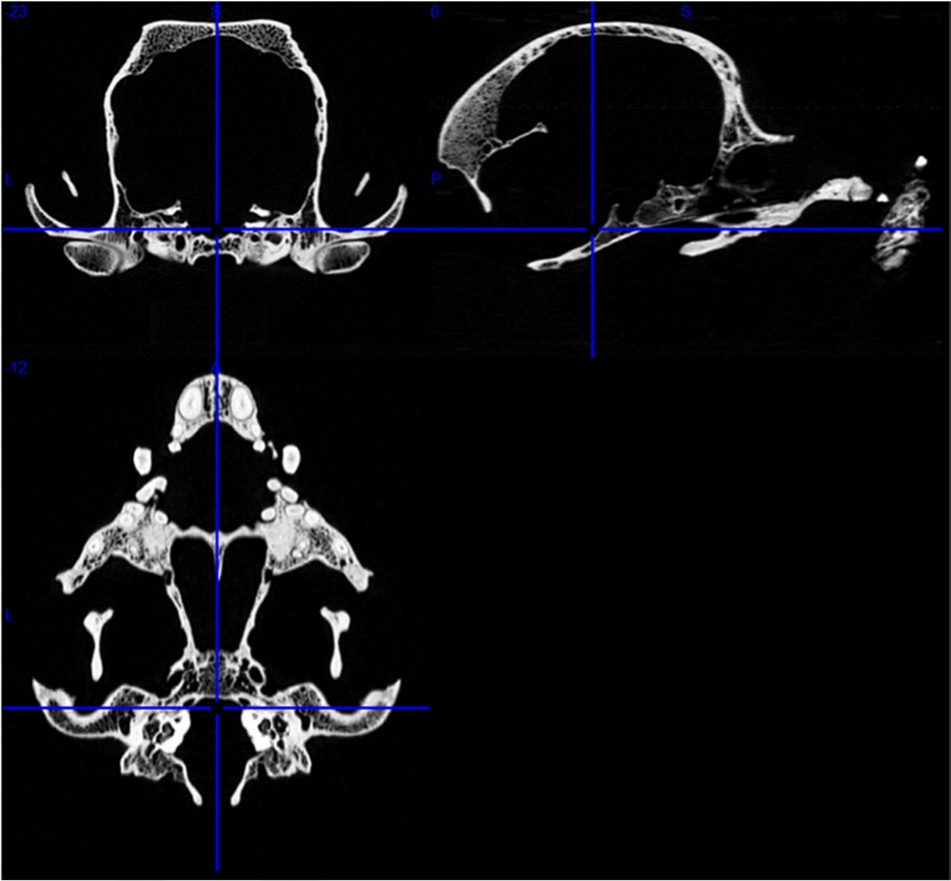
Skull of a Boston terrier on a CT image series. First row: transverse and sagittal views. Second row: dorsal view.

Skulls were transported to Moritz Kaposi Teaching Hospital Dr. Jozsef Baka Diagnostic, Radiation Oncology, Research and Teaching Center (Medicopus Nonprofit Ltd.) for the scanning procedure. We used a medical CT device (Siemens Somatom Definition AS+ CT, Siemens, Erlangen, Germany), and we selected a setting on the scanner that provided the best image quality with an Ultra High Resolution (UHR) protocol. Acquisition parameters were set as: 200 mAs exposure, 140 kV tube voltage, 16 × 0.6 mm collimation, and spiral data collection with 0.85 pitch factor to digitize the bones (Fig. 1). The name of this specific Siemens’ examination setting protocol is “UHR,” which stands for “ultra-high resolution”. This setting is applied to small examination areas such as the human inner ear and optic nerve imaging. Using this setting resulted in a longer imaging duration and higher radiation exposure, but considering the technical possibilities, it produced the best possible contrast ratios and spatial resolution. The benefit of using the medical CT with the UHR option was the time optimization for scanning a large number of specimens. In contrast to using an industrial micro-CT scanner, where the scanning process of one skull takes about 40–45 minutes (including scanning and reconstruction), the speed of the medical CT was approximately 5-6 minutes per skull. In some cases, a piece of polyurethane foam was placed under the skull (below the basioccipital bone) to stabilize and straighten its position during scanning.

### Image reconstruction and conversion

Overlapping axial scans were reconstructed from the collected raw data using the Siemens Somaris/7 Syngo CT software program (ver. VA48A) with v80u convolution kernel and osteo window (very sharp). The resolution of the images was pixel size 0.3 × 0.3 mm, slice thickness 0.6 mm and reconstruction increment 0.3 mm. The images were archived in DICOM (Digital Imaging and Communications in Medicine) format. Further image acquisition and reconstruction parameters can be found in text files located in the ‘DICOM headers’ folder of the shared dataset. Afterwards, the multislice volumes were converted to one NIfTI (Neuroimaging Informatics Technology Initiative) file per subject (resulting in a smaller file size due to the conversion but maintaining the image quality). Each file name was constructed based on the data listed in Table S1 to aid easier navigation and selection. The first three numbers refer to the species ID, the A-B-C characters refer to the sex (A: male, B: female, C: unknown), and the last 3-4 characters are the short breed identifier (ID). When multiple species with the same breed and sex occurred, an increasing counting number was added to the sex ID. These three IDs in the file name are separated with underscores. For example, the 026_A_BORD.nii.gz file refers to a male border collie. NIfTI-files are located in the ‘Skull volumes’ folder of the shared data.

### Volume cropping and viewport save

**Fig. 2:**
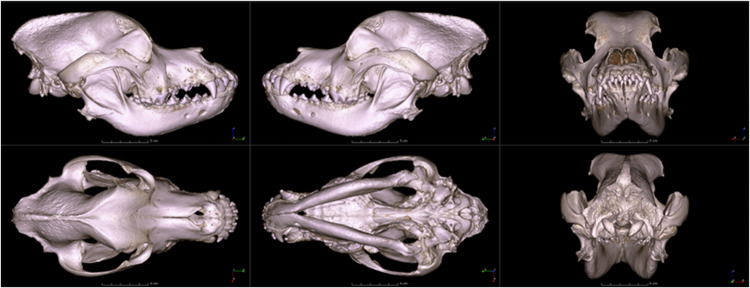
Skull of a Saint Bernard dog from different views. First row: right, left, and front views. Second row: top, bottom, and back views.

To generate an overview of the image volumes (without the necessity to download them for inspection of suitability), a further post-processing step was carried out. Volumes were cropped to remove extraneous surrounding regions (the space around the skulls), and screenshots were generated from six primary perspectives of the volume-rendered CT image series, resulting in left, right, front, back, top, and bottom views (Fig. 2). All of these images were created using 3D Slicer^25^, which can be found in the ‘Thumbnail images’ folder of the dataset.

## Data Records

The dataset is available on FigShare^26^, where skull volumes, DICOM headers and thumbnail image files can be downloaded in zip-archive files. It is also available on ELTE’s Cloud server (https://nc.elte.hu/s/doZnwPjgeBaZ7as), where files can be downloaded individually. Both repositories contain a summary table .xls file with the Individual IDs, Species/Breed IDs, Sex, Species, Latin species names, Breed, and Breed abbreviations (also attached as Table S1), and a video illustrating the database (Video S1). The video is also available through this link: https://www.youtube.com/watch?v=wE5elULOfWk.

## Technical Validation

Every image was carefully examined by a skilled CT operator (TD) and two veterinary anatomists (KC, ÖP,) to ensure that the image quality meets the desired standards, it is devoid of artefacts and possesses adequate contrast for the purpose of image analysis. The whole process was carried out according to the ISO 9001:2015 quality management system and ISO 14001:2015 environmental management system.

### Supplementary information

Canid and felid skull CT database video

Table S1

## Data Availability

No custom code has been used for this study.
